# Extracting functionally feedforward networks from a population of spiking neurons

**DOI:** 10.3389/fncom.2012.00086

**Published:** 2012-10-19

**Authors:** Kathleen Vincent, Joseph S. Tauskela, Jean-Philippe Thivierge

**Affiliations:** ^1^School of Psychology, University of OttawaOttawa, ON, Canada; ^2^Synaptic Therapies and Devices Group, National Research Council, Institute for Biological SciencesOttawa, ON, Canada

**Keywords:** neuronal avalanches, cortical culture, Poisson model, functional connectivity, feedforward networks

## Abstract

Neuronal avalanches are a ubiquitous form of activity characterized by spontaneous bursts whose size distribution follows a power-law. Recent theoretical models have replicated power-law avalanches by assuming the presence of functionally feedforward connections (FFCs) in the underlying dynamics of the system. Accordingly, avalanches are generated by a feedforward chain of activation that persists despite being embedded in a larger, massively recurrent circuit. However, it is unclear to what extent networks of living neurons that exhibit power-law avalanches rely on FFCs. Here, we employed a computational approach to reconstruct the functional connectivity of cultured cortical neurons plated on multielectrode arrays (MEAs) and investigated whether pharmacologically induced alterations in avalanche dynamics are accompanied by changes in FFCs. This approach begins by extracting a functional network of directed links between pairs of neurons, and then evaluates the strength of FFCs using Schur decomposition. In a first step, we examined the ability of this approach to extract FFCs from simulated spiking neurons. The strength of FFCs obtained in strictly feedforward networks diminished monotonically as links were gradually rewired at random. Next, we estimated the FFCs of spontaneously active cortical neuron cultures in the presence of either a control medium, a GABA_A_ receptor antagonist (PTX), or an AMPA receptor antagonist combined with an NMDA receptor antagonist (APV/DNQX). The distribution of avalanche sizes in these cultures was modulated by this pharmacology, with a shallower power-law under PTX (due to the prominence of larger avalanches) and a steeper power-law under APV/DNQX (due to avalanches recruiting fewer neurons) relative to control cultures. The strength of FFCs increased in networks after application of PTX, consistent with an amplification of feedforward activity during avalanches. Conversely, FFCs decreased after application of APV/DNQX, consistent with fading feedforward activation. The observed alterations in FFCs provide experimental support for recent theoretical work linking power-law avalanches to the feedforward organization of functional connections in local neuronal circuits.

## Introduction

Spontaneous activity accounts for a significant proportion of the brain's energy consumption (Raichle, 2006) and serves important roles both in the development of neural circuits (Blankenship and Feller, 2010) and in the modulation of neuronal responses to external stimuli (Arieli et al., 1996). A hallmark of spontaneous activity is its high degree of spatial and temporal organization. In a broad spectrum of circuits, spontaneous activity alternates between periods of relative quiescence interleaved with short bursts of activity that recruit a spatially delimited population of neurons. In one regime of activity, these bursts follow a power-law distribution, in which bursts recruiting a small number of neurons occur markedly more often than larger bursts (Beggs and Plenz, 2003). This characteristic distribution of bursts, termed a neuronal avalanche, has been reported in several systems, including dissociated hippocampal cultures (Tang et al., 2008), somatosensory cortical slices (Gireesh and Plenz, 2008), and *in vivo* (Petermann et al., 2009). A power-law distribution of avalanches is indicative of neural dynamics with no characteristic scale and is conjectured to form an optimal state for information processing, computation, and learning (Shew et al., 2011).

**Figure 1:**
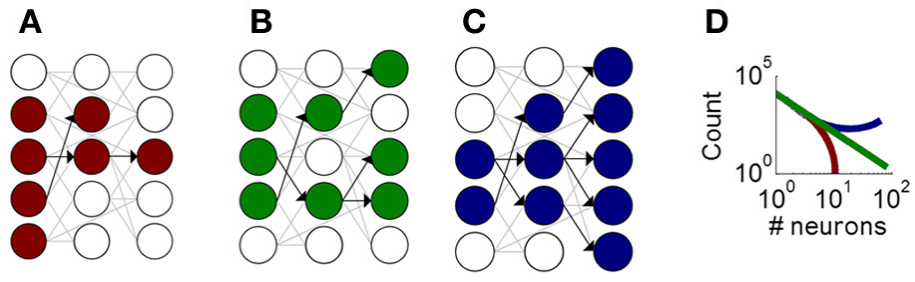
**Illustration of the critical branching model producing neuronal avalanches.** Schematic neurons (circles) connected by synapses (gray and black links). Neurons activated during an avalanche are shown in color. Black links show functional feedforward connections that represent the directed influence of one neuron on another. **(A)** A fading avalanche, where fewer and fewer neurons become activated across functional layers. **(B)** A sustained avalanche, where a comparable number of neurons are activated across layers. **(C)** An amplifying avalanche, where increasingly more neurons become activated across layers. **(D)** Theoretical distribution of avalanche sizes for fading (red), sustained (green), and amplifying (blue) avalanches.

Despite the role of neuronal avalanches in information processing, their origins remain unclear. One theoretical account of avalanches is based on a branching process, whereby groups of neurons activate each other in a feedforward manner (Figure 1) (Haldeman and Beggs, 2005). This account distinguishes between three classes of avalanches, with either fading activity across layers (Figure 1A), sustained activity across layers (Figure 1B), or amplifying activity across layers (Figure 1C). These three classes of avalanches lead to distinct distributions of avalanche sizes that capture experimentally derived distributions (Figure 1D).

In apparent contradiction with a feedforward branching model, both experimental (Beggs and Plenz, 2003) and theoretical (Pajevic and Plenz, 2009; Benayoun et al., 2010; Rubinov et al., 2011) work show that power-law avalanches can arise in massively recurrent networks. To account for this result, a “functional feedforward hypothesis” has been proposed (Benayoun et al., 2010). Accordingly, the functional connectivity of a network (i.e., its pattern of neuronal interactions) possesses a prominent feedforward drive even in the midst of strong recurrent projections.

The possibility of functionally feedforward connections (FFCs) being expressed by a recurrent network has been raised by different theoretical accounts (Goldman, 2009; Murphy and Miller, 2009). Yet, it is unclear whether living systems rely on FFCs to generate neuronal avalanches. To address this question, we monitored spontaneous activity from cultured cortical neurons plated on multielectrode arrays (MEAs) (Marom and Shahaf, 2002; Beggs and Plenz, 2003; Wagenaar et al., 2006). We pharmacologically altered the distribution of neuronal avalanches and computed the effect on the strength of FFCs by using a method of Schur decomposition. If the feedforward hypothesis of avalanches holds, alterations in the distribution of avalanche sizes should be accompanied by systematic changes to the strength of FFCs, providing empirical support for a theory-grounded link between functional connectivity and avalanche dynamics.

## Materials and methods

### Cultured cortical neurons on microelectrode arrays

**Figure 2:**
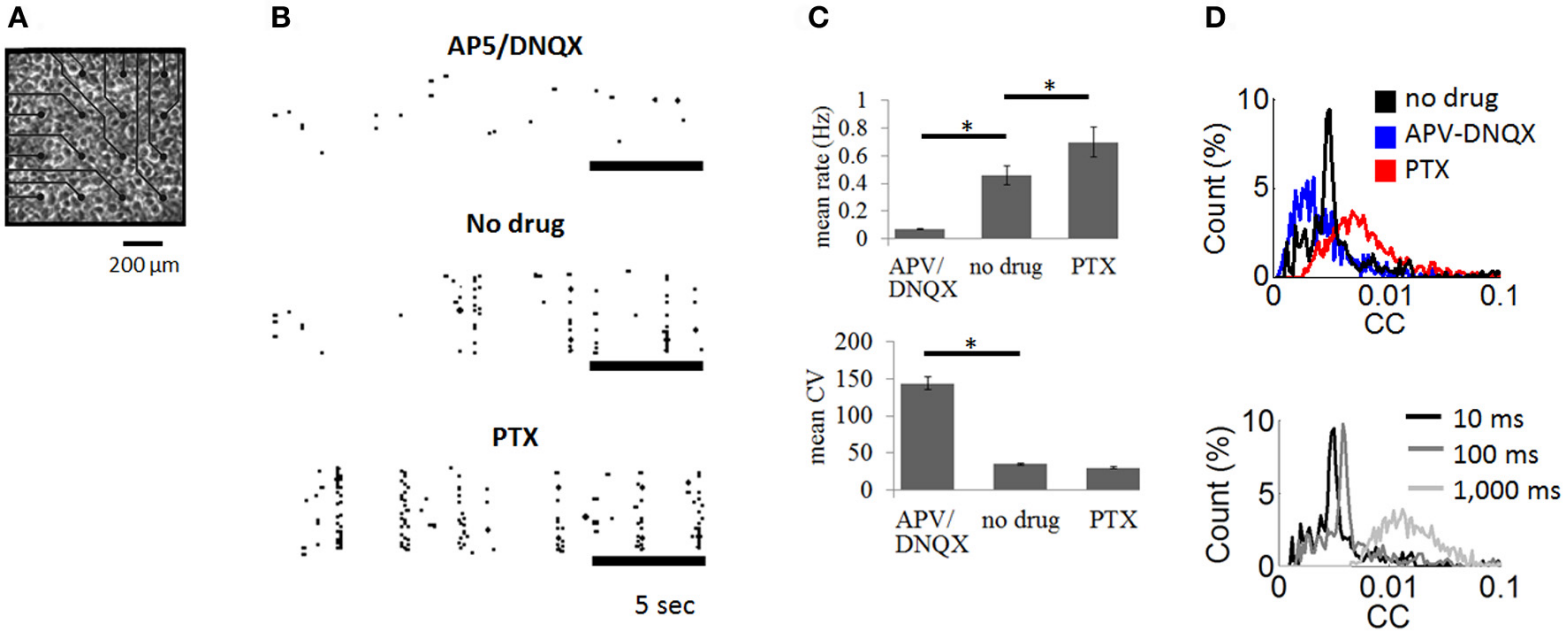
**Cortical cultures plated on multielectrode arrays. (A)** Culture of cortical neurons plated on multielectrode array (only a subset of array shown). **(B)** Examples of spike rasters with bath application of APV/DNQX or PTX as well as control recordings with no drug. **(C)** Mean firing rate (upper panel) and coefficient of variation (lower panel) of spontaneous activity across experimental conditions. Vertical bars are SEM. ^*^ = *p* < 0.001, paired sample *t*-test. **(D)** Top: distribution of pairwise cross-correlations obtained for all pairs of neurons in the no drug, APV-DNQX, and PTX conditions. Bottom: cross-correlations obtained for windows of different sizes (10 ms, 100 ms, 1,000 ms) using all pairs of neurons from the no drug condition.

Culturing and plating of primary cortical neurons was performed as previously described (Tauskela et al., 2008). Briefly, cell cultures were recorded using 60 electrodes placed in an 8 × 8 array, with electrodes absent at the corners (ALA Scientific, Germany) (Figure 2A). Dissociated primary cortical neurons were prepared from 18 day prenatal Sprague Dawley rats (Charles River, St. Constant, QC, Canada). Cells were filtered through 40μm cell strainer and were plated on poly-ethylinimine-coated MEAs at 1.5 million cells per ml of medium in an MEA. Plating medium consisted of MEM supplemented to 25 mM glucose, 10% fetal calf serum, 10% horse serum and 1% penicillin in streptomycin. MEAs were maintained in a humidified incubator at 37°C and a 5% carbon dioxide/95% air atmosphere, and osmolality was strictly controlled. To control the growth of glia, a mitotic inhibitor (15μg/ml of 5-fluoro-2′-deoxyuridine and 35μg/ml of uridine) was added to each culture at 4 days *in vitro* (DIV). A 50% media change was performed once a week with MEM containing 10% horse serum. Each culture subjected to recording was inspected to ensure that neurons exhibited a dense, homogenous monolayer with <5% death. All recordings were performed at 14–19 DIV, an age for which cultures have sufficiently matured to produce maximal firing rates and number of active channels (Novellino and Zaldivar, 2010).

### Pharmacology

Agents were added directly to the medium of cultures. Control (no drug) recordings were performed within 1 h of drug application. Two separate drug conditions were studied in different cultures: (1) GABA_A_ receptor antagonist picrotoxin (PTX); and (2) NMDA receptor antagonist (2R)-amino-5-phsphonovaleric acid (APV) combined with AMPA receptor antagonist 6,7-dinitroquinoxaline-2,3-dione (DNQX). Drugs were prepared from stock solution. Final bath concentrations were 5μM PTX, 2μM DNQX, and 20μM APV. A wash-in of 10 min preceded all drug recordings.

### Spontaneous activity

Recordings were performed using Multi Channel System (MCS, Reutlingen, Germany) MC Rack software for microelectrode arrays (Musick, 2008). MEAs were mounted on the recording platform and capped with a sterile vented tissue culture lid to maintain sterility. Prior to each recording, MEAs were given a 20-min incubation period on the platform to equilibrate within an incubator maintained at 37°C with 5% carbon dioxide. Each recording was carried out for 20 min duration. Recording parameters were as follows: 1100.0 amplifier gain, input voltage range of −2048 to +2048 mV and a sampling frequency of 5000 Hz. Low frequency signals were removed using a high pass filter with a cut-off frequency of 200 Hz.

### Spike sorting

Online extracellular spike detection was performed using MCS software. Spikes were detected online using a 3 standard deviation (s.d.) threshold above mean using MC_RACK. The resulting spike data from MCS were stored for offline spike sorting, performed with Plexon software (version 3.0, Plexon Inc., Texas USA). Spike data were analyzed using custom software in Matlab software (Mathworks Inc., Natick, MA). Paired *t*-tests were employed to compare mean spike rates and mean coefficients of variation (CV, standard deviation of spike trains over mean rate) across experimental conditions. These tests were performed after pooling all neurons within each condition (control, APV/DNQX, PTX). Neurons whose firing rate was ±5 s.d. above or below the mean of all neurons within a condition were excluded from statistical analysis.

### Neuronal avalanches

We identified avalanches by using non-overlapping time bins of a fixed size. This time bin was set by default to 10 ms (we also examined variations in the length of the time bin, see “Results”). This duration was chosen to reflect the timescale over which coordinated activity likely affects the response of downstream neurons (Cohen and Kohn, 2011); alternative methods could also be explored (Shimazaki and Shinomoto, 2007). An avalanche is defined as a series of consecutive bins where all bins have at least one spike. In addition, an avalanche must be preceded and followed by at least one time bin without spikes. The size of an avalanche is defined as the total number of neurons activated from the start to finish of the avalanche. Aside from avalanche sizes, other aspects of multi-electrode recordings can follow a power-law; we refer readers to other work for in-depth analyses (Beggs and Plenz, 2003; Gireesh and Plenz, 2008; Petermann et al., 2009).

In order to determine the goodness-of-fit of a power-law distribution to avalanches sizes, we employed a maximum likelihood approach (Newman, 2005; Touboul and Destexhe, 2010). Distributions that follow a power-law function can be described as follows:

(1) $$p\left(x\right)=Cx^{-\alpha },$$

where *p*(*x*) is the probability of observing an avalanche of a given size *x* (the number of active neurons), α is a scaling exponent that defines the slope of the power-law distribution, and *C* is a constant term.

The maximum likelihood estimate of α, denoted $\hat{\alpha }$, is provided by (assuming a continuous underlying power-law distribution for simplicity):

(2) $$\hat{\alpha }=1+n{\left[\sum_{i=1}^{n}\log\frac{x_{i}}{x_{\min}}\right]}^{-1},$$

where *i* = 1 … *n* are the observed values of *x*. Depending on the nature of the data, it may sometimes be necessary to set *x*_min_ to a particular range of distribution; however, by visual inspection we did not find a particular justification for excluding parts of the distribution, so we set *x*_min_ to the minimum value of the avalanche size distributions.

Statistical error in the estimation of α is given by:

(3) $$\text{error}=\frac{\hat{\alpha }-1}{\sqrt{n}}.$$

### Contiguity index

To estimate whether avalanches propagated in a wave-like manner across the MEAs, we computed a contiguity index corresponding to the fraction of spikes that are preceded by spikes on one or more nearest-neighbor neuron (i.e., neurons either from the same electrode or immediately adjacent on the array). More formally, we denote the status of a neuron *i* at time *t* as *s*_*i*_ (*t*) = 1 if a spike is emitted, and *s*_*i*_ (*t*) = 0 otherwise. We index the set of neighboring neurons as *m* = 1, …, *M*. For each given spike (*s*_*i*_(*t*) = 1), we compute:

(4) $$h_{i}(t)=\{\text{​}\begin{array}{ll} 1, & \sum_{m}s_{m}(t-1)>0\\ 0, & \text{otherwise}, \end{array}$$

and obtain the contiguity index of neuron *i* as follows:

(5) $$\text{Contiguity index}\left(i\right)=100\times \;\frac{\sum_{t}h_{i}\left(t\right)}{\sum_{t}s_{i}\left(t\right)}.$$

If activity propagated in a strict wave, nearly 100% of all activity would arise from nearest neighbors; by comparison, neuronal avalanches typically show a contiguity index of less than 30% (Beggs and Plenz, 2003).

### Critical branching

We sought to quantify the propagation of activity in neuronal avalanches. Using an established procedure, we computed the branching parameter σ, defined as the average number of neurons that are activated as descendants when one ancestor neuron is activated (Beggs and Plenz, 2003). In recordings of spontaneous activity, the branching parameter σ corresponds to the average number of neurons activated in a given time bin (during an avalanche), given a single neuron being activated in the preceding time bin. The average value of σ for an entire recording is given by:

(6) $$\sigma =\sum_{d=0}^{n_{\max}}d\times p(d),$$

where *p*(*d*) is the probability of observing *d* descendants, *n*_max_ is the maximal number of active neurons, and *d* is the number of neuron descendants. These descendants are computed as:

(7) $$d=\text{round}\left(\frac{n_{d}}{n_{a}}\right)\text{​},$$

where *n*_*a*_ is the number of neuron ancestors observed in the first time bin, *n*_*d*_ is the number of active neurons in the second time bin of an avalanche, and round is a rounding operator to the nearest integer. We estimated *p*(*d*) (the probability of observing *d* descendants) as follows:

(8) $$p(d)=\sum_{\text{avalanches}}\left(\frac{n\sum_{a|d}}{n\sum_{a}}\right)\left(\frac{n_{\max}-1}{n_{\max}-n_{a}}\right),$$

where *n*_∑_*a*|*d*__ is the total number of spikes in all avalanches when *n*_*d*_ descendants were observed and *n*_∑_*a*__ is the total number of ancestors observed in all avalanches.

### Pairwise cross-correlations

We calculated the cross-correlation between all pairs of neurons. We began by downsampling the timeseries of activity for each neuron by dividing the total recording time into non-overlapping windows of fixed duration. A default duration of 10 ms was employed, consistent with the timescale over which correlated activity likely affects the response of downstream neurons (Cohen and Kohn, 2011). For each neuron, we then represented every window by a single value of 0 (no spike emitted) or 1 (at least one spike emitted). Once the activity of all neurons was downsampled in this way, we computed the cross-correlation between a given pair of neurons as follows:

(9) $$CC_{ij}=\frac{E\left\{[i-E\left(i\right)]\left[j-E\left(j\right)\right]\right\}}{\sqrt{E\left\{{\left[i-E\left(i\right)\right]}^{2}\right\}E\left\{{\left[j-E\left(j\right)\right]}^{2}\right\}}},$$

where *i* and *j* are the time-series of two neurons having means *E*{*i*} and *E*{*j*}, respectively.

### Directed functional connectivity

We constructed a graph of functional connectivity between all pairs of spontaneously active neurons on the array. In a subsequent step (described below) this graph will serve as a basis for estimating the strength of FFCs amongst neurons. There are two requirements for generating this graph: (1) it must be weighted (connections must have continuous values); and (2) it must be directed (connections between pairs of neurons must point in a particular direction of influence from one electrode to another and may not be symmetrical).

Several measures fulfill the above criteria, including Granger causality (Cadotte et al., 2008), Bayesian approaches (Pajevic and Plenz, 2009), and partial directed coherence (Gourevitch et al., 2006). We settled on the use of transfer entropy (TE), recently shown to be useful in reconstructing the functional architecture of a network from its ongoing dynamics (Gourevitch and Eggermont, 2007; Vicente et al., 2011). TE quantifies the amount of information in a neuron found in the past history of another neuron. It can be used to probe asymmetries in neural relations (i.e., neuron A influencing B, but B not influencing A) (Schreiber, 2000). These asymmetries allow the evaluation of both feedback and feedforward contributions to network activity.

The amount of TE from neuron *j* to neuron *i* (measured in bits) is given by:

(10) $$TE_{j\to i}=\sum p\left(i_{t+1},i_{t},j_{t}\right)\log_{2}\frac{p\left(i_{t+1}|i_{t},j_{t}\right)}{p\left(i_{t+1}|i_{t}\right)},$$

where *i*_*t*_ denotes the status of neuron *i* at time *t*, and could be either 1 or 0, indicating a spike or no spike, respectively, and *p* denotes the empirical probability of having the status denoted in parentheses. First, *p*(*i*_*t*+1_, *i*_*t*_, *j*_*t*_) denotes the joint probability of an event involving {*i*_*t*+1_ = 1, *i*_*t*_ = 1, *j*_*t*_ = 1}, obtained empirically as the count of all such events divided by the total length of the recording in ms. Second, *p*(*i*_*t*+1_|*i*_*t*_, *j*_*t*_) denotes a conditional probability, obtained by summing all events involving {*i*_*t*+1_ = 1, *i*_*t*_ = 1, *j*_*t*_ = 1}, and dividing by the sum of events involving {*i*_*t*+1_ = 0, *i*_*t*_ = 1, *j*_*t*_ = 1} and events involving {*i*_*t*+1_ = 1, *i*_*t*_ = 1, *j*_*t*_ = 1}. Finally, *p*(*i*_*t*+1_|*i*_*t*_) is obtained by summing all events involving {*i*_*t*+1_ = 1, *i*_*t*_ = 1} and dividing by the sum of events involving {*i*_*t*+1_ = 0, *i*_*t*_ = 1} and events involving {*i*_*t*+1_ = 1, *i*_*t*_ = 1}. The sum in Equation 10 is taken over all combinations of *i*_*t*+1_, *i*_*t*_, and *j*_*t*_. Details of the implementation of the algorithm are beyond the scope of this paper and can be found elsewhere along with free software (Ito et al., 2011). Taken as a whole, Equation 10 describes how the future activity of a neuron is influenced by its own past history, thereby making TE mostly (although not completely) independent of firing rates of both neurons *i* and *j* considered.

Because of potential errors in the estimation of TE, we performed a bootstrap reshuffling of spike times. This bootstrapping was performed for each neuron independently across its entire recording time. The activity of a given neuron was converted to a binary vector of “1” and “0” denoting the presence and absence of spikes respectively. This vector was randomly shuffled by taking each spike and the inter-spike interval immediately following it, and moving it to a different location on the vector. This method of shuffling preserves the distributions of both spike rates and inter-spikes intervals. The shuffling procedure was repeated 100 times, and a value of TE for shuffled data (*TE*^bootstrap^_*j*→*i*_) was obtained by averaging the values of TE obtained across all repeats of the shuffling procedure. The final value of TE from neuron *j* to *i* (*TE*^final^_*j*→*i*_) was obtained by subtracting the value of TE obtained from shuffled data (*TE*^bootstrap^_*j*→*i*_) from the value of TE obtained from the original data (*TE*^data^_*j*→*i*_), and normalizing by the entropy rate (*H*_*i*_) (the conditional entropy of neuron *i* conditional on its past):

(11) $$TE_{j\to i}^{\text{final}}=\frac{TE_{j\to i}^{\text{data}}-TE_{j\to i}^{\text{bootstrap}}}{H_{i}},$$

where the entropy rate is given by:

(12) $$H_{i}=-\sum p\left(i_{t+1},i_{t}\right)\log_{2}p\left(i_{t+1}|i_{t}\right).$$

To account for estimation errors in computing TE, we analytically derived the variance of TE (Equation 10) as follows:

(13) $$\begin{array}{c} V_{j\to i}=\frac{1}{T}\sum p\left(i_{t+1},i_{t},j_{t}\right)\left(1-p\left(i_{t+1},i_{t},j_{t}\right)\right)\\ \;\times {\left[\log_{2}\left(\frac{p\left(i_{t+1}|i_{t},j_{t}\right)}{p\left(i_{t+1}|i_{t}\right)}\right)-TE_{j\to i}\right]}^{2}\text{​}, \end{array}$$

where *T* is the temporal precision employed for calculating TE (set by default to 2 ms; time-lags between 1–3 ms did not substantially alter results).

In order to generate a graph of functional connectivity, we computed TE values (Equation 11) and the variance of *TE*^data^_*j*→*i*_ (Equation 13) for all pairs of neurons in a given recording. Then, we eliminated (by setting to zero) all connections where *TE*^data^_*j*→*i*_ was less than 3 s.d. above *TE*^bootstrap^_*j*→*i*_, as well as all negative values of *TE*^final^_*j*→*i*_. The final graph could be expressed as an *N* × *N* matrix *W* (where *N* is the number of neurons) of directed and weighted functional connections where all entries are either positive or zero.

### Functionally feedforward connectivity

Once the above-defined graph of functional connectivity was obtained, the strength of FFCs was estimated. These connections reflect the degree to which the activation of groups of neurons follows a feedforward chain (Figure 1). We estimated FFCs by performing a Schur decomposition of the functional connectivity matrix *W* (defined above) (Goldman, 2009; Murphy and Miller, 2009). The Schur decomposition extracts orthogonal eigenvectors of the *W* matrix of functional connectivity, revealing eigenmodes that send both feedback and feedforward projections amongst themselves. This method offers an advantage over traditional eigenvalue decomposition, as the latter can estimate recurrent modes but not feedforward modes of activity.

For a given matrix *W* of functional connectivity, we obtained the following decomposition:

(14) $$W=UMU^{-1},$$

where *U* is a unitary matrix with orthogonal columns, and *M* is an upper triangular matrix. To quantify the strength of FFCs (denoted *f*^*M*^), we computed the sum of absolute squares of the off-diagonal elements of *M*, and normalized by the sum of absolute squares of all the elements of *M* (Murphy and Miller, 2009):

(15) $$f^{M}=\left(\text{Tr}\left({\text{MM}}^{†}\right)-\sum_{\alpha }{\left|\beta_{\alpha }^{\text{M}}\right|}^{2}\right)/\text{Tr}\left({\text{MM}}^{†}\right),$$

where M^†^ is the conjugate transpose of *M*, Tr is the trace, and β^M^_α_ are the eigenvalues of *M*. In Equation 15, *f*^M^ is interpreted as the proportion of ongoing dynamics that are driven by FFCs. This value is complementary to the proportion of functionally *recurrent* connections, such that the sum of feedforward and recurrent connections adds up to 1.0. While the Schur decomposition of a matrix *M* is not unique, the absolute squares of all elements of *M* are unitary invariant. The eigenvalues of *M* are also unitary invariant. The estimation of FFCs relies strictly upon Equation 15, which is unitary invariant and therefore does not depend upon the solution obtained by a given Schur decomposition. Statistical comparisons of FFCs between experimental conditions (no drug, APV/DNQX, and PTX) were carried out with a paired sample *t-test* after pooling all neurons within each condition and excluding values of FFC that were ±5 s.d. above or below the mean of FFCs within a condition.

### Poisson model

We tested the ability of Schur decomposition to estimate the strength of FFCs in a synthetic dataset of spikes generated from a population of Poisson neurons. The firing rate of these neurons could be modulated by interactions with neighboring neurons. Neuronal interactions were controlled by an *N* × *N* matrix *P* of transition probabilities (i.e., the probability that a spike in one neuron at time *t* will generate a spike in another neuron at time *t* + 1), where *N* is the total number of neurons in the population. The matrix *P* hence defined all directed influences of a given neuron on another neuron. For simplification, these values remained fixed over time.

The probability of a neuron spiking at a given time-step *t* was influenced by both the Poisson process and the transition matrix *P* (Roxin et al., 2008). The probability ${\hat{p}}_{i}$ that a neuron *i* will become active at a given time-step *t* is:

(16) $${\hat{p}}_{i}=\frac{v_{i}-\left(1-v_{i}\tau_{r}\right)\sum_{j}v_{j}p_{ij}}{1-v_{i}\tau_{r}},$$

where τ_*r*_ is a refractory period and *p*_*ij*_ are elements of the *P* transition matrix. We simulated spiking activity at a resolution of 1 kHz, and set *v* = 0.0001 to yield an approximate Poisson firing rate of 0.1 Hz for all *N* = 100 neurons. The actual firing rate of neurons could be higher than that value depending on the *P* transition matrix.

## Results

### Functionally feedforward connections in simulated data

Our first step was to examine whether Schur decomposition can estimate the strength of FFCs in a simulated network of Poisson spiking neurons. In these simulations, we manipulated the matrix *P* of transition probabilities by gradually shifting from a strictly feedforward network to a highly recurrent/random network, and using Schur decomposition to estimate the proportion of dynamics that are driven by FFCs.

**Figure 3:**
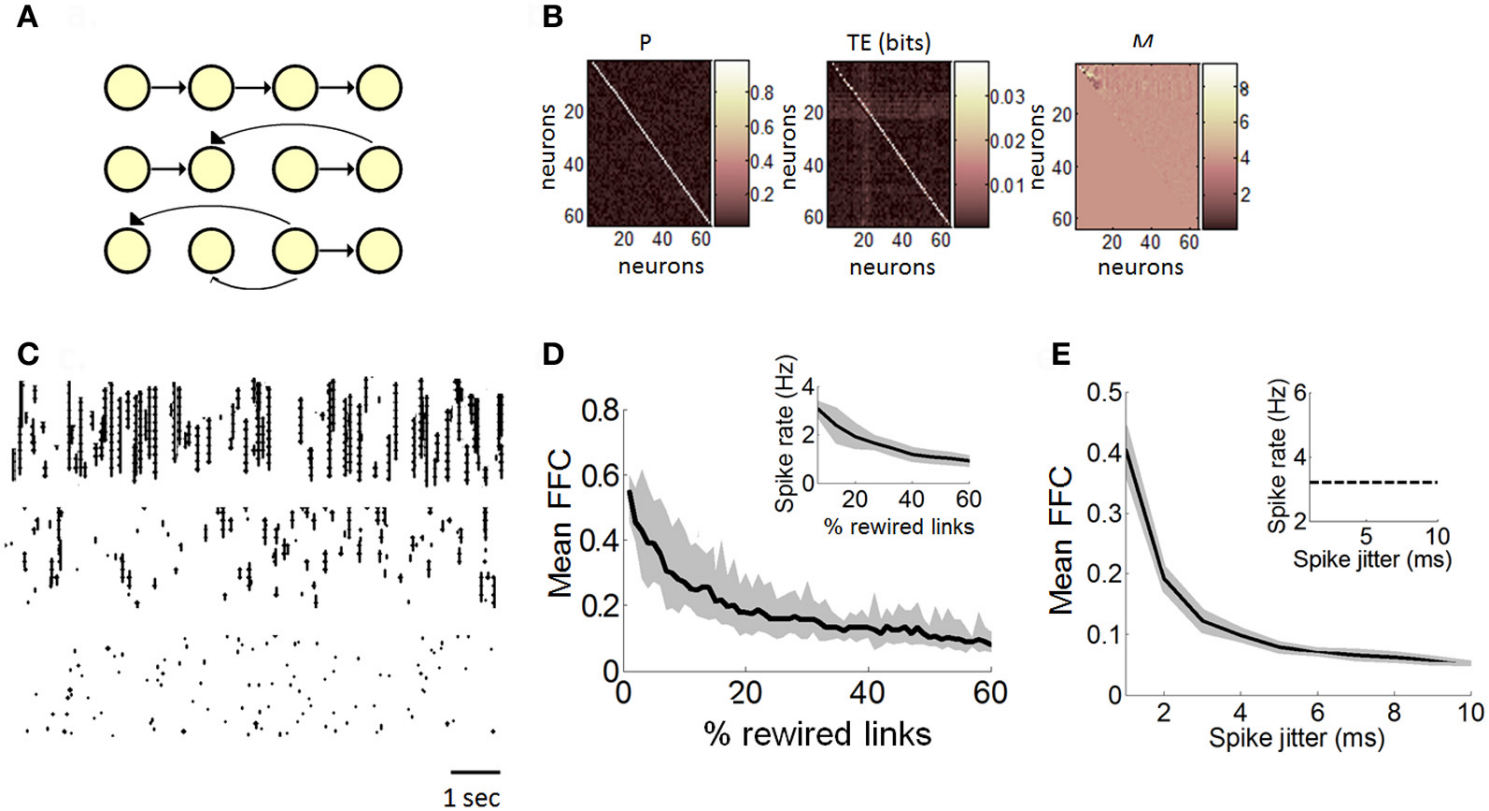
**Estimation of functionally feedforward connectivity in a Poisson model. (A)** Simulated feedforward network of spiking neurons (yellow nodes) connected by synapses (black arrows). A strictly feedforward network (top panel) is gradually rewired (middle and bottom panel), resulting in progressively more recurrent projections. **(B)** Matrix of transition probabilities (*P*), transfer entropy (TE), and Schur decomposition (*M*) for a strictly feedforward network (e.g., panel “**A**,” top panel). **(C)** Examples of spike rasters generated with a Poisson model with either 100% (top panel), 50% (middle panel), or 10% (bottom panel) feedforward transition probabilities. **(D)** The mean proportion of functionally feedforward connectivity (FFC) diminishes gradually as the number of randomly rewired links increases. Inset: spike rates decrease as the percentage of rewired links increases. **(E)** In a strictly feedforward network, the mean FFC diminishes as the magnitude of spike jittering increases. Inset: spike jittering has no effect on mean spike rates. **(D–E)** Solid black line: mean values taken from 100 independent runs of the Poisson model. Gray area: SEM.

We began with a feedforward network representing transition probabilities that are strictly feedforward (Figure 3A, top; only a subset of the *N* = 100 neurons are shown for ease of visualization). In this network, the presence of a link between two neurons indicates a transition probability of *p*_*ij*_ = 1.0, while the absence of a link indicates *p*_*ij*_ = 0.0. We generated 5 min of activity with this strictly feedforward model, and stored all spikes over time. We then computed a graph of directed functional connectivity based on TE (Equation 11), and performed a Schur decomposition to estimate the strength of feedforward connectivity (FFC) (Equations 14, 15). We found a striking similarity between the *P* matrix and the matrix of functional connectivity obtained by TE (Figure 3B), showing that TE captured the statistics of interactions amongst pairs of neurons in the model.

We performed 100 independent runs of the feedforward model and obtained a mean FFC proportion of 0.58 (s.d. 0.04). We compared this value to different versions of the model where transition probabilities were gradually rewired by choosing a link at random and changing its receiving neuron (Figure 3A, middle and bottom graphs; see Figure 3C for examples of rasters). The proportion of dynamics driven by FFCs diminished gradually as networks shifted from a feedforward to recurrent connectivity (Figure 3D). Schur decomposition was thus sensitive to transition probabilities in the dynamics of the Poisson model, and provided a monotonically decreasing relation between the degree of rewiring and the proportion of feedforward drive, with a strictly feedforward network yielding the strongest feedforward drive. These simulations provide a proof-of-principle that Schur decomposition can distinguish neural dynamics on the basis of FFCs.

Next, we examined the robustness of FFCs to the precision of spikes in a strictly feedforward network. We generated 5 min of spiking activity from a network of 100 Poisson neurons with strictly feedforward transition probabilities (Figure 3A, top). We randomly disrupted the temporal precision of spikes using a method of spike jitter and computed the effect on the strength of FFCs. The spike jitter method involves randomly moving spikes of individual neurons to different positions in time. A pre-determined window of Δ*t* ms is set such that spikes can be shuffled by a maximum value of Δ*t* ms either forwards or backwards in time. In different simulations, we tested different magnitudes of spike jitter, from minimal jitter (where spikes were randomly shifted by 1 ms at most from their original position in time) to maximal jitter (where spikes were shifted by up to 10 ms). Spike jitter decreased the mean FFCs, while maintaining mean spike rates constant (Figure 3E). Hence, the strength of FFCs can be influenced not only by the proportion of FFCs in the model (Figure 3C), but also by spike precision in the propagation of feedforward activity. Furthermore, these results show that FFCs can be modulated independently of firing rates (Figure 3E, inset). Next, we turn to experimental data in order to relate the dynamics of neuronal avalanches to the strength of functionally feedforward networks.

### Neuronal avalanches in cortical networks

We recorded spontaneous activity from cortical cultures and extracted neuronal avalanches of spiking activity (see “Materials and Methods”). Three conditions were compared: (1) no-drug controls (*N* = 6); (2) AMPA receptor antagonist combined with NMDA receptor antagonist (APV/DNQX) (*N* = 5); and (3) GABA_A_ receptor antagonist (PTX) (*N* = 5). Spontaneous activity in control recordings was characterized by bursts of synchronized activity interspersed by periods of relative quiescence (Figure 2B, middle panel). Bath application of APV/DNQX abolished synchronized activity (Figure 2B, top panel), while PTX increased its occurrence (Figure 2B, bottom panel). APV/DNQX cultures had a lower spike rates and a higher CV than controls. PTX cultures yielded the opposite effect, with higher firing rates and lower CVs (Figure 2C). Pairwise cross-correlations (see “Materials and Methods”) were lower for APV/DNQX cultures compared to controls (Wilcoxon rank-sum test, *P* < 0.00015) and comparatively higher for PTX cultures (*P* < 0.021) (Figure 2D, top). All distributions had a nonzero mean consistent with *in vitro* activity (Beggs and Plenz, 2003). Increasing the window employed for cross-correlations from 10 to 100 ms did not substantially alter the resulting distribution; however, further increasing the window to 1,000 ms yielded a broad and diffuse distribution (Figure 2D, bottom).

**Figure 4:**
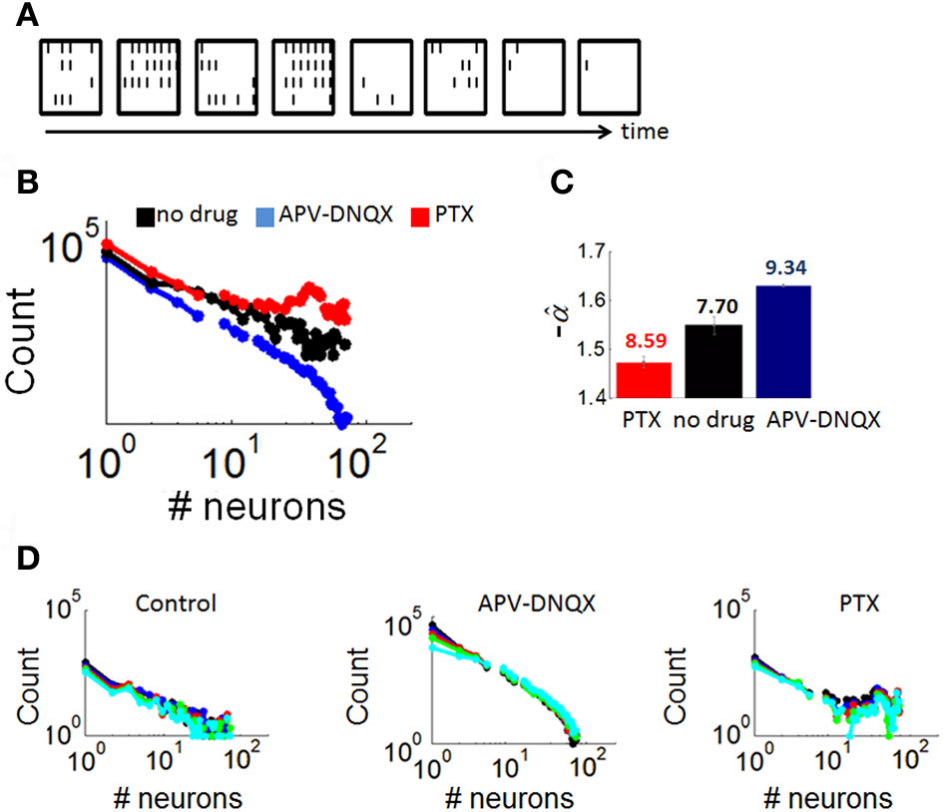
**Distributions of neuronal avalanches modulated by pharmacology. (A)** Example of an avalanche obtained from a control culture. Each frame corresponds to 10 ms of activity. Black lines correspond to the presence of at least one spike in each of the 64 channels of the array (configured in a 8 × 8 spatial arrangement). **(B)** Average distribution of avalanche size for cultures with either no drug treatment, APV/DNQX, or PTX. **(C)** Estimation of the slope of best-fitting power-law ($\hat{\alpha }$) for distributions in panel “**A**,” obtained with maximum likelihood (see “Materials and Methods”). Numbers above each bar indicate log likelihood values. Vertical bars are statistical errors (Equation 3). **(D)** Distributions of avalanche sizes (as in panel **A**) with different bin sizes (window of time used for the computation of avalanches). Bin sizes: black = 1 ms; blue = 2 ms; red = 4 ms; green = 8 ms; cyan = 16 ms.

We extracted neuronal avalanches from all recordings (see “Materials and Methods”; Figure 4A shows a representative avalanche). Over all cultures, the total number of avalanches obtained was 41,544 (no drug), 4224 (APV/DNQX), and 34,632 (PTX). We employed a contiguity index to verify that avalanches did not propagate in a strictly wave-like manner (Beggs and Plenz, 2003) (see “Materials and Methods”). In controls, an average 23.64% (s.d. 18.61) of spikes were preceded by a spike on at least one nearest-neighbor electrode. Similar values were obtained for PTX (28.57%, s.d. 21.59) and APV/DNQX (17.29%, s.d. 9.77) cultures. Hence avalanches did not activate a substantial proportion of nearest neighbors on the array, suggesting that the propagation of avalanches did not proceed in a wave-like manner.

Next, we examined whether avalanches led to the reactivation of precise spike patterns. Here, a spike pattern is defined as the subset of neurons (i.e., at least two neurons) that fire within a given timeframe. We counted the percentage of timeframes where a spike pattern was reactivated at a later time during the same avalanche. Reactivated spike patterns accounted for less than 1% percent of activity in all conditions (controls, 0.44%; PTX, 0.7%; APV/DNQX, 0.12%). Thus, neuronal avalanches were characterized predominantly by a feedforward progression through a series of individual spike patterns rather than a recurrent loop that reactivates a given subset of patterns.

Distributions of avalanche sizes (number of active neurons) in all three conditions (Figure 4B) were fitted with a power-law using maximum likelihood estimation (see “Materials and Methods”) (Figure 4C). Neuronal avalanches in controls followed a power-law distribution with a slope of α = −1.55 (statistical error: 0.02), a characteristic of dynamics that are poised between completely randomized and completely synchronized activity (Beggs and Plenz, 2003). Avalanches in PTX recordings were characterized by a double peak, due to a prominence of avalanches recruiting a large proportion of neurons on the array. These avalanches were fitted with a slope of α = −1.47 (statistical error: 0.04), higher than that of controls. Finally, avalanches in APV/DNQX followed a power-law with a slope of α = −1.63 (statistical error: 0.01), lower than that of controls and typical of dynamics with highly synchronized activity. Distributions of avalanche sizes were not markedly altered in analyses where we modified the size of time bins (Figure 4D), showing robustness of results to changes in that parameter.

### Functional networks of cortical networks

**Figure 5:**
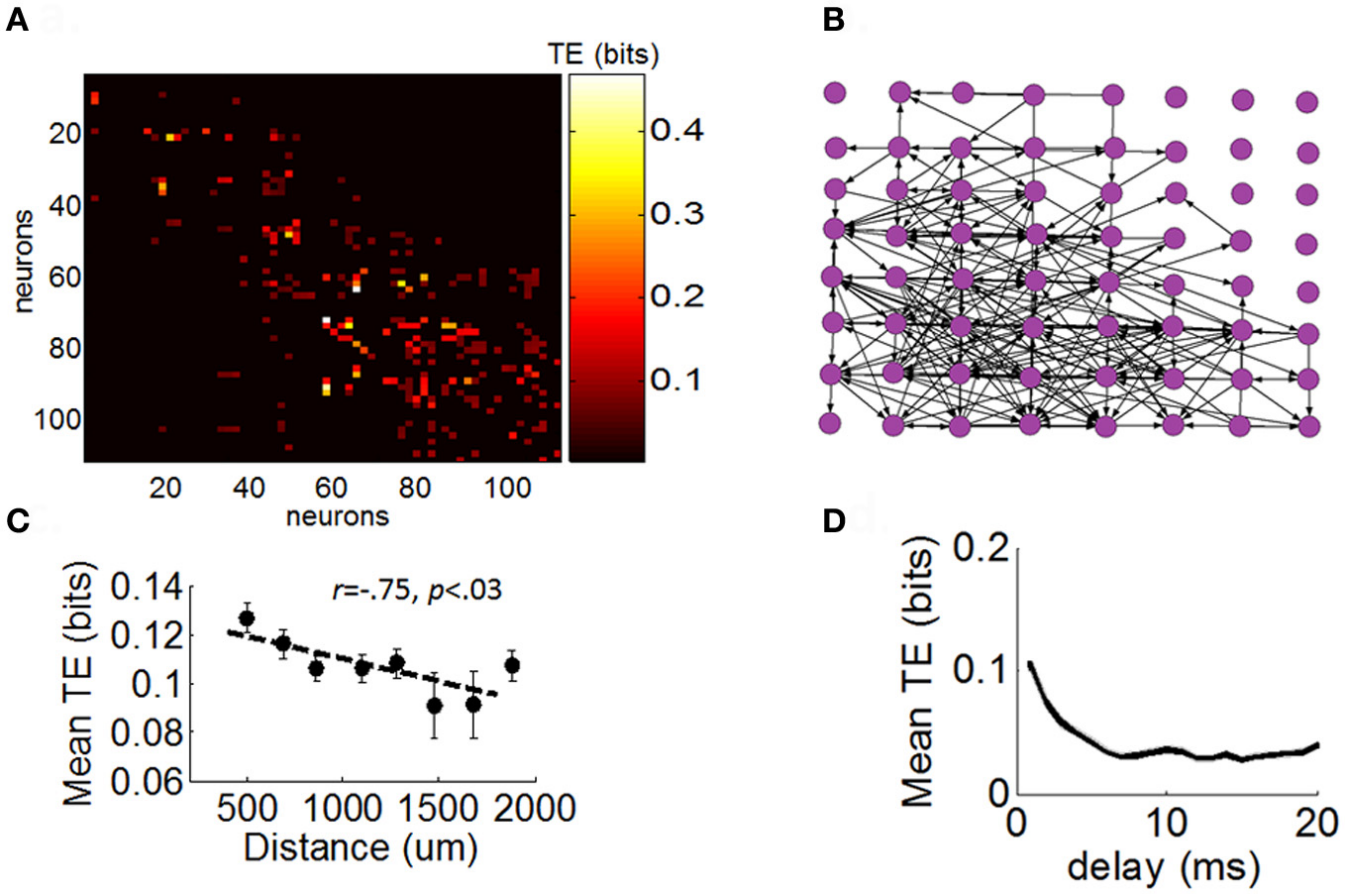
**Directed functional network obtained with transfer entropy. (A)** Matrix of functional connectivity obtained with transfer entropy (see “Materials and Methods”). **(B)** Positive values of transfer entropy from the matrix in panel “**A**” are converted to directed links (black arrows) between electrodes of the multielectrode arrays. For illustration purposes, we represent only a single neuron per channel of the array. **(C)** Relation between transfer entropy and physical distance between electrodes on the array. Each dot represents the mean TE obtained for a particular physical distance on the array (averaged across all arrays and pairs of neurons). Dashed line: best-fitting linear regression. Vertical bars: SEM. **(D)** Relation between temporal delay between spikes and values of transfer entropy. Shows mean TE obtained for particular delays (Equation 10). Shaded area: SEM.

Next, we sought to determine the extent to which the different distributions of neuronal avalanches described above were related to the strength of FFCs. We began by constructing a network of functional connectivity for each array using TE (see “Materials and Methods”) (Figure 5A). This network quantifies the amount of information (in bits) transmitted from one given neuron to another. For illustration purposes, we registered a graph of TE across all pairs of neurons to a 2D spatial array reflecting the relative position of electrodes with which each neuron is recorded (Figure 5B). This graph presents a highly recurrent network of functional connections, from which it is difficult to determine the contribution of FFCs without further analysis. The amount of TE between pairs of neurons was related to their spatial distance on the array (Figure 5C) and was strongest for short temporal delays between spikes (Figure 5D).

### Functionally feedforward connections in cortical networks

**Figure 6:**
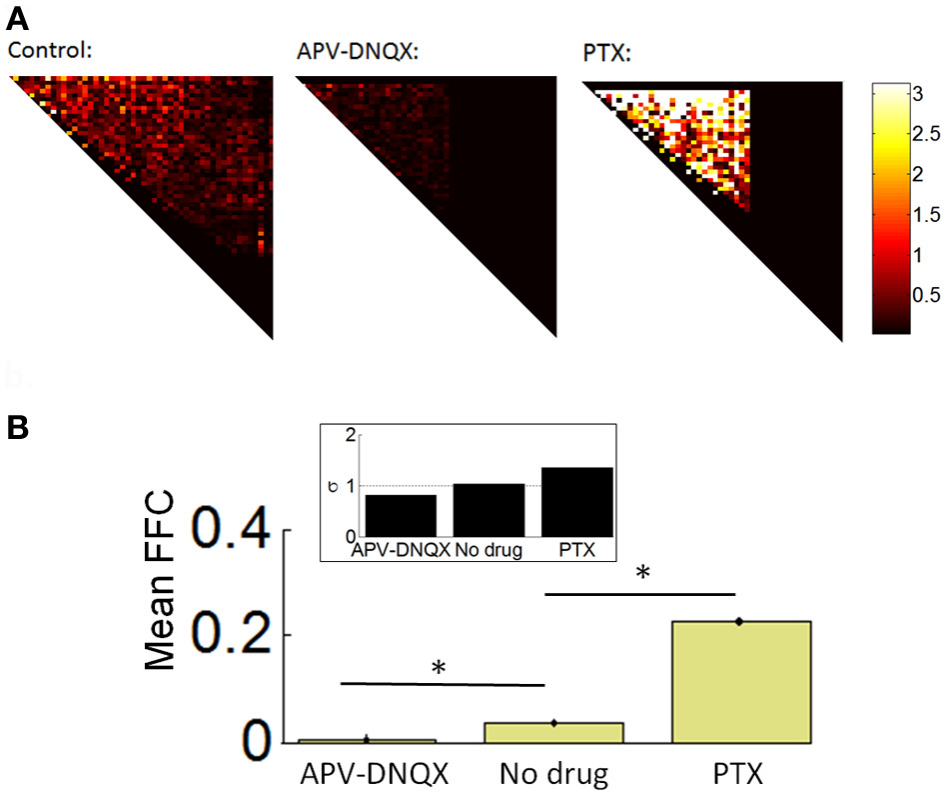
**Schur decomposition of functional networks. (A)** Examples of *M* matrices obtained from Schur decomposition (see “Materials and Methods”), providing an estimate of the strength of functionally feedforward connectivity. For clarity, only the upper triangular elements are shown. **(B)** Mean proportion of functionally feedforward connectivity (FFC) obtained from Equation 15. ^*^p < 0.0001 (paired sample *t*-test). Inset: mean critical branching parameter. Dashed line: σ = 1.

We analyzed the strength of FFCs using a Schur decomposition of the TE matrix *W* obtained for all pairs of neurons on a given array (see “Materials and Methods”). The Schur decomposition *W* = *UMU*^−1^ yields an upper triangular matrix *M* from which we estimated the strength of functionally FFCs. Examples of *M* matrices are shown in Figure 6A, along with the mean strength of FFCs across all arrays (Figure 6B). The strength of FFCs for control recordings was significantly higher than for APV/DNQX recordings [*t*_(782)_ = 12.24, *p* < 0.0001] and significantly lower than for PTX recordings [*t*_(773)_ = 14.65, *p* < 0.0001]. These results show that the strength of FFCs is modulated by pharmacology. Taken together with distributions of neuronal avalanches (Figures 4B–C), results suggest that APV/DNQX leads to fading avalanches accompanied by a reduction in the strength of FFCs when compared to controls. On the other hand, PTX leads to amplifying avalanches accompanied by an increase in the strength of FFCs. Hence the distribution of avalanches and the strength of FFCs were concomitantly modulated by pharmacology.

We compared the above results with the mean branching parameter of controls and pharmacologically treated networks (see “Materials and Methods”). No-drug networks attained a mean branching parameter near 1.0, indicative of sustained avalanche dynamics (Beggs and Plenz, 2003). APV/DNQX and PTX networks attained higher and lower values, respectively (Figure 6B, inset). The branching parameter provides corroborative evidence that the strength of FFCs is altered by pharmacology, with fading avalanches under APV/DNQX and amplifying avalanches under PTX.

### Robustness to noise and length of recordings

**Figure 7:**
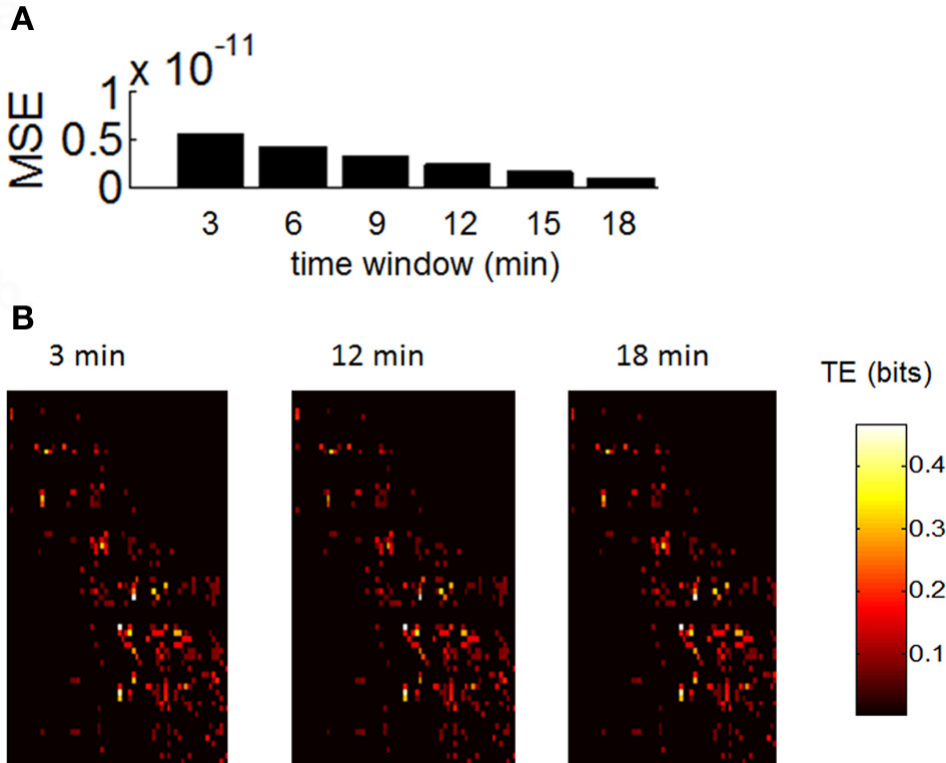
**Robustness of transfer entropy to the size of the recording period. (A)** Mean square error (MSE) between values of transfer entropy obtained for consecutive windows of 3, 6, …, 18 min. **(B)** Example of transfer entropy obtained over all pairs of neurons during a single recording. Similar matrices are generated with recording periods of 3, 12, and 18 min.

In order to assess whether our estimation of functional networks was sensitive to the duration of recordings, we took spontaneous activity from control recordings and computed TE across all pairs of neurons for time windows of 3, 6, 9, 12, 15, and 18 min (Figure 7). We then computed the mean squared error in TE between values obtained for each time window and values obtained for the total recording length (20 min). The error decreased monotonically as we increased the length of the time window from 3 to 18 min (Figure 7A). In addition, the overall appearance of TE between all pairs of neurons was largely similar between shorter and longer time windows (Figure 7B). Based on these results, we would not expect that lengthening our recording windows beyond 20 min would lead to drastic changes in TE. However, we caution that the presence of non-stationarities on a long time-scale may play a role (Sasaki et al., 2007); this is beyond the scope of our current work.

**Figure 8:**
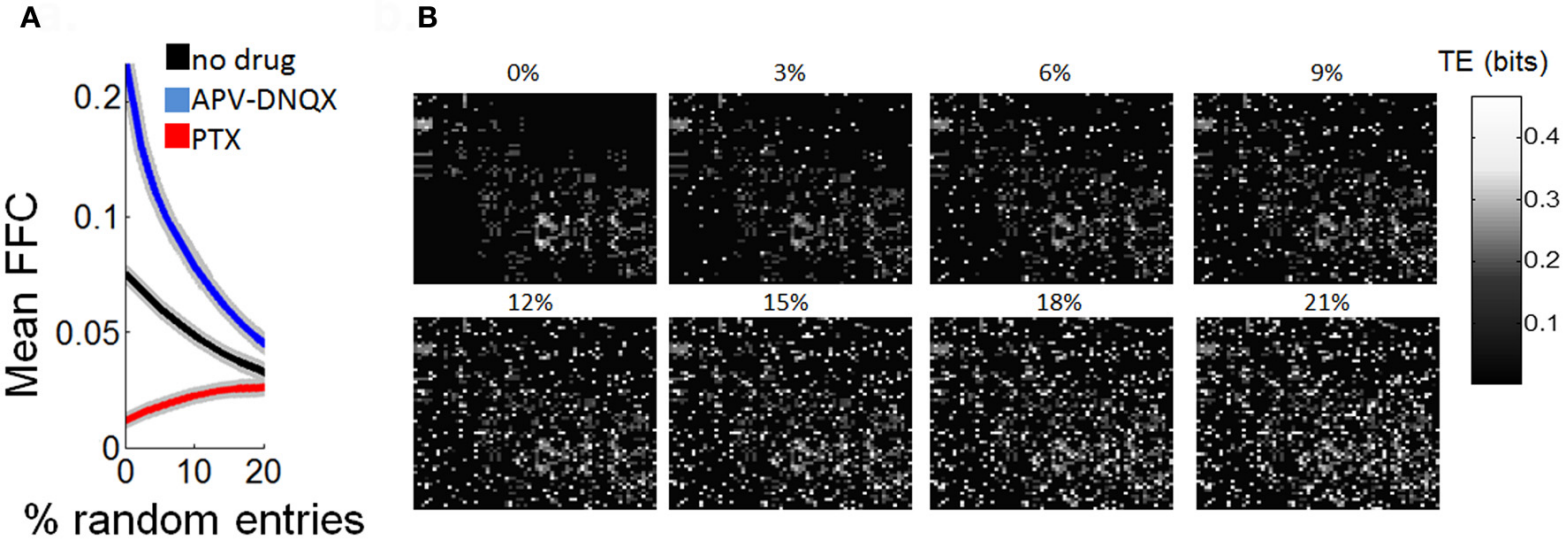
**Estimation of functionally feedforward strength is robust to partial randomization of transfer entropy. (A)** Gradual decline in mean strength of functionally feedforward connections (FFCs) as random entries (values of 0.5 bits) are added to a matrix of transfer entropy. Shaded area is SEM. **(B)** Illustration of a transfer entropy matrix where random entries are gradually added. The % of random entries (over all possible entries in the matrix) is shown above each panel.

In a final series of analyses, we examined the robustness of Schur decomposition when adding noise to the functional connectivity obtained by TE. Starting from functional networks of the control condition, we gradually added functional connections at random (values of 0.5 bits). As random connections were added, the mean strength of FFCs decreased gradually. Approximately 20% of random connections were required to decrease the mean strength of FCCs by half. Adding random connections made the network gradually more recurrent, and it is thus expected that the strength of FFCs should decrease accordingly. We performed the same analysis with functional networks treated with APV/DNQX and PTX. The mean strength of FFCs for drug-treated networks gradually converged toward the mean of control networks as random connected were added. Yet, with 20% of randomly added connections it was still possible to distinguish between the three experimental conditions based on the strength of their FFCs (Figures 8A–B).

## Discussion

While cortical neurons are part of a highly recurrent network, strong feedback excitation is typically balanced by strong feedback inhibition (van Vreeswijk and Sompolinsky, 1998; Higley and Contreras, 2006). While the functional consequences of this balanced regime remain to be elucidated, recent theoretical work suggests that it may facilitate the emergence of patterns of activity that evolve over time in a functionally feedforward manner (Murphy and Miller, 2009). Here, we developed a novel method to estimate the proportion of neuronal activity in a multi-neuron recording that is driven by a functionally feedforward network. This method constructs a functional network of weighted and asymmetrical interactions between all pairs of neurons in a given data set (Gourevitch and Eggermont, 2007; Vicente et al., 2011), followed by extraction of feedforward and feedback modes of activity through Schur decomposition (Goldman, 2009).

We first tested the method on artificial data generated from a Poisson model where firing rate was influenced by neighboring neurons. These highly simplified phenomenological networks allowed examination of feedforward information propagation in both strictly feedforward and highly recurrent circuitry. Estimates of FFC diminished monotonically in simulations that gradually increased the proportion of recurrent connections, showing an ability to track the strength of FFCs on the basis of spiking activity. Simulations were also sensitive to the precision of activity propagation, with random perturbations in the spike timing of feedforward propagation leading to a lower estimate of FFCs.

Next, we applied the method of FFC estimation to multielectrode recordings of spontaneous activity in cortical cultures. In these cultures, activity is characterized by avalanches whose size distribution follows a power-law consistent with sustained activation (Beggs and Plenz, 2003). These avalanches were altered pharmacologically to yield either fading avalanches (APV/DNQX) or amplifying avalanches (PTX) (Shew et al., 2011). We computed the strength of FFCs in control and drug-treated cultures and showed that, compared to control cultures, APV/DNQX-treated cultures had significantly lower FFCs. In PTX-treated cultures, on the other hand, the strength of FFCs was significantly higher than that of control cultures.

The above results can be understood in terms of functional feedforward activation (Figures 1A–C). Accordingly, the feedforward propagation of activity under APV/DNQX fades more rapidly than in controls, leading to a shift in the distribution of avalanche sizes (Figure 1D). Conversely, in PTX-treated cultures, the feedforward propagation of activity amplifies over time, moving the distribution of avalanche sizes in the opposite direction. Importantly, this functionally feedforward account describes the propagation of activity in a network, and not its pattern of structural connections. Even highly recurrent networks, despite their feedback connections, can—depending on their configuration—propagate activity in a feedforward manner (Goldman, 2009).

We propose that FFCs underlie the propensity of recurrent cortical circuits to propagate activity in a chain-like feedforward manner. Neuronal avalanches, despite arising from highly recurrent circuits both *in vivo* (Petermann et al., 2009) and *in vitro* (Gireesh and Plenz, 2008), show hallmark features of feedforward activation. In previous work, under no circumstances was an electrode immediately (or even 4 timeframes later) reactivated during avalanche activity (Beggs and Plenz, 2003). Further, our own results show that less than 1% of spike patterns generated during avalanches were repeats of a pattern activated previously during the same avalanche. These results show that avalanche dynamics are characterized by a forward progression through a series of non-repeating patterns, as opposed to a recurring leitmotif of activity that cycles through a fixed repertoire.

There are several advantages of functionally feedforward networks compared to their recurrent counterpart. Low feedback in functionally feedforward networks prevents runaway excitation. Furthermore, functionally feedforward networks are optimal at sustaining memory traces of inputs (Ganguli et al., 2008), and respond with a longer timecourse than recurrent networks (Murphy and Miller, 2009), thus allowing for a greater diversity of responses (Goldman, 2009).

Our proposal of a relationship between FFC and neuronal avalanches is supported by a body of theoretical work. One account based on a branching model captures avalanche distributions by assuming a feedforward chain of propagation (Beggs and Plenz, 2003). In this model, sustained activation is produced when neurons activated at one given time-step activate a similar number of neurons at the subsequent step during an avalanche. In another model, a power-law distribution of avalanches is produced by a functionally feedforward architecture that activates groups of neurons in a defined order despite being embedded in a highly recurrent circuitry (Benayoun et al., 2010). In the latter model, simulations of fully connected as well as sparsely connected networks can both produce avalanche distributions that follow a power-law—a determining factor being the strength of FFC. Finally, simulations of a reduced model show that recurrent networks can behave as “feedforward networks in disguise” provided that they are derived from the Schur modes of a feedforward network (Goldman, 2009).

Recent theoretical work proposes that neuronal avalanches can emerge from simulated networks with balanced excitation and inhibition (Poil et al., 2012). Such a link between balanced activity and neuronal avalanches is consistent with experimental work where pharmacological disruption in the ratio of excitation and inhibition alters the power-law distribution of avalanches (Shew et al., 2011). Other work, however, seems inconsistent with a link between balanced activity and neuronal avalanches. In a balanced regime, cross-correlations between pairs of neurons are near zero (Ecker et al., 2010; Renart et al., 2010), which is incompatible with *in vitro* neuronal avalanches where correlations are in the 0–0.2 range (Beggs and Plenz, 2003). Near-zero correlations depend on the nature of the preparation, the anatomical region considered, the firing rate of neurons, and the spike sorting method employed (Cohen and Kohn, 2011). These considerations leave open the question of how balanced activity could relate to avalanches.

Our proposed approach to estimate FFCs offers an interesting perspective for relating FFCs to the strength of cross-correlations between pairs of neurons. A potential relation between FFCs and cross-correlations is suggested by our findings relating APV-DNQX to a downward shift in the distribution of correlations (Figure 2D). This shift leaves largely unaltered the tail of the distribution. PTX treatment, on the other hand, leads to an upward shift as well as a broadening in the distribution of correlations. These findings offer only a preliminary step in relating FFCs to cross-correlations, a challenging question that we leave for future empirical and theoretical work.

While our analyses of cortical activity highlight a systematic relation between FFCs and dynamics, our conclusions are limited by some factors. Recordings from finite tissue do not allow us to grasp the full extent of activation propagation beyond a few orders of magnitude. In addition, the method by which we evaluate FFCs from experimental data contains several steps where estimation error can be introduced (spike sorting, directed networks, Schur decomposition) and would benefit from a more streamlined approach. Finally, experiments do not allow us to selectively manipulate the strength of FFCs and examine their impact on neuronal activity. To address this issue, biophysically detailed simulations of neuronal circuits could investigate how pharmacological treatment alters the balance of feedforward and feedback excitation in recurrent circuits. Moving in that direction, some models show a link between synaptic strengths and avalanche dynamics (Levina et al., 2007; Rubinov et al., 2011). It remains unclear, however, how different rules for synaptic plasticity (e.g., spike timing-dependent plasticity, STDP) can reshape a circuit and generate patterns of neural dynamics consistent with a functionally feedforward network. While some work suggests that STDP leads to the natural emergence of feedforward patterns in recurrent networks (Jun and Jin, 2007; Liu and Buonomano, 2009; Fiete et al., 2010), other work shows conflicting results (Kunkel et al., 2011).

In conclusion, our work highlights a systematic relation between functionally feedforward networks and neuronal avalanche. Despite limitations imposed by the proposed method and the nature of the recordings, our results provide empirical support for a functionally feedforward hypothesis of avalanche dynamics as a basis for neuronal avalanches. Accordingly, highly recurrent cortical circuits can propagate activity in a strictly feedforward manner, thus showing that a given cortical circuit may support a broad range of behaviors and respond in a flexible manner to computational demands.

### Conflict of interest statement

The authors declare that the research was conducted in the absence of any commercial or financial relationships that could be construed as a potential conflict of interest.

## References

[B1] Arieli A. Sterkin A. Grinvald A. Aertsen A. (1996). Dynamics of ongoing activity: explanation of the large variability in evoked cortical responses. Science 273, 1868–1871 10.1016/j.neures.2009.02.011 8791593

[B2] Beggs J. M. Plenz D. (2003). Neuronal avalanches in neocortical circuits. J. Neurosci. 23, 11167–11177 14657176 10.1523/JNEUROSCI.23-35-11167.2003 PMC6741045

[B3] Benayoun M. Cowan J. D. Van Drongelen W. Wallace E. (2010). Avalanches in a stochastic model of spiking neurons. PLoS Comput. Biol. 6:e1000846 10.1371/journal.pcbi.1000846 20628615 PMC2900286

[B4] Blankenship A. G. Feller M. B. (2010). Mechanisms underlying spontaneous patterned activity in developing neural circuits. Nat. Rev. Neurosci. 11, 18–29 10.1038/nrn2759 19953103 PMC2902252

[B5] Cadotte A. J. Demarse T. B. He P. Ding M. (2008). Causal measures of structure and plasticity in simulated and living neural networks. PLoS ONE 3:e3355 10.1371/journal.pone.0003355 18839039 PMC2556387

[B6] Cohen M. R. Kohn A. (2011). Measuring and interpreting neuronal correlations. Nat. Neurosci. 14, 811–819 10.1038/nn.2842 21709677 PMC3586814

[B7] Ecker A. S. Berens P. Keliris G. A. Bethge M. Logothetis N. K. Tolias A. S. (2010). Decorrelated neuronal firing in cortical microcircuits. Science 327, 584–587 10.1126/science.1179867 20110506

[B8] Fiete I. R. Senn W. Wang C. Z. Hahnloser R. H. (2010). Spike-time-dependent plasticity and heterosynaptic competition organize networks to produce long scale-free sequences of neural activity. Neuron 65, 563–576 10.1016/j.neuron.2010.02.003 20188660

[B9] Ganguli S. Huh D. Sompolinsky H. (2008). Memory traces in dynamical systems. Proc. Natl. Acad. Sci. U.S.A. 105, 18970–18975 10.1073/pnas.0804451105 19020074 PMC2596211

[B10] Gireesh E. D. Plenz D. (2008). Neuronal avalanches organize as nested theta- and beta/gamma-oscillations during development of cortical layer 2/3. Proc. Natl. Acad. Sci. U.S.A. 105, 7576–7581 10.1073/pnas.0800537105 18499802 PMC2396689

[B11] Goldman M. S. (2009). Memory without feedback in a neural network. Neuron 61, 621–634 10.1016/j.neuron.2008.12.012 19249281 PMC2674525

[B12] Gourevitch B. Bouquin-Jeannes R. L. Faucon G. (2006). Linear and nonlinear causality between signals: methods, examples and neurophysiological applications. Biol. Cybern. 95, 349–369 10.1007/s00422-006-0098-0 16927098

[B13] Gourevitch B. Eggermont J. J. (2007). Evaluating information transfer between auditory cortical neurons. J. Neurophysiol. 97, 2533–2543 10.1152/jn.01106.2006 17202243

[B14] Haldeman C. Beggs J. M. (2005). Critical branching captures activity in living neural networks and maximizes the number of metastable States. Phys. Rev. Lett. 94, 058101 10.1103/PhysRevLett.94.058101 15783702

[B15] Higley M. J. Contreras D. (2006). Balanced excitation and inhibition determine spike timing during frequency adaptation. J. Neurosci. 26, 448–457 10.1523/JNEUROSCI.3506-05.2006 16407542 PMC6674406

[B16] Ito S. Hansen M. E. Heiland R. Lumsdaine A. Litke A. M. Beggs J. M. (2011). Extending transfer entropy improves identification of effective connectivity in a spiking cortical network model. PLoS ONE 6:e27431 10.1371/journal.pone.0027431 22102894 PMC3216957

[B17] Jun J. K. Jin D. Z. (2007). Development of neural circuitry for precise temporal sequences through spontaneous activity, axon remodeling, and synaptic plasticity. PLoS ONE 2:e723 10.1371/journal.pone.0000723 17684568 PMC1933597

[B18] Kunkel S. Diesmann M. Morrison A. (2011). Limits to the development of feed-forward structures in large recurrent neuronal networks. Front. Comput. Neurosci. 4:160 10.3389/fncom.2010.00160 21415913 PMC3042733

[B19] Levina A. Herrmann J. M. Geisel T. (2007). Dynamical synapses causing self-organized criticality in neural networks. Nat. Phys. 3, 857–860

[B20] Liu J. K. Buonomano D. V. (2009). Embedding multiple trajectories in simulated recurrent neural networks in a self-organizing manner. J. Neurosci. 29, 13172–13181 10.1523/JNEUROSCI.2358-09.2009 19846705 PMC6665184

[B21] Marom S. Shahaf G. (2002). Development, learning and memory in large random networks of cortical neurons: lessons beyond anatomy. Q. Rev. Biophys. 35, 63–87 11997981 10.1017/s0033583501003742

[B22] Murphy B. K. Miller K. D. (2009). Balanced amplification: a new mechanism of selective amplification of neural activity patterns. Neuron 61, 635–648 10.1016/j.neuron.2009.02.005 19249282 PMC2667957

[B23] Musick K. (2008). Three-Dimensional Microelectrode Array for Recording Dissociated Neuronal Cultures. M.S., University of Illinois at Urbana-Champaign. 10.1039/b820596e 19568672 PMC2818679

[B24] Newman M. E. J. (2005). Power laws, Pareto distributions and Zipf's law. Contemp. Phys. 46, 323–351

[B25] Novellino A. Zaldivar J.-M. (2010). Recurrence quantification analysis of spontaneous electrophysiological activity during development: characterization of *in vitro* neuronal networks cultured on multi electrode array chips. Adv. Artif. Intell. 2010, 1–10

[B26] Pajevic S. Plenz D. (2009). Efficient network reconstruction from dynamical cascades identifies small-world topology of neuronal avalanches. PLoS Comput. Biol. 5:e1000271 10.1371/journal.pcbi.1000271 19180180 PMC2615076

[B27] Petermann T. Thiagarajan T. C. Lebedev M. A. Nicolelis M. A. Chialvo D. R. Plenz D. (2009). Spontaneous cortical activity in awake monkeys composed of neuronal avalanches. Proc. Natl. Acad. Sci. U.S.A. 106, 15921–15926 10.1073/pnas.0904089106 19717463 PMC2732708

[B28] Poil S. S. Hardstone R. Mansvelder H. D. Linkenkaer-Hansen K. (2012). Critical-state dynamics of avalanches and oscillations jointly emerge from balanced excitation/inhibition in neuronal networks. J. Neurosci. 32, 9817–9823 10.1523/JNEUROSCI.5990-11.2012 22815496 PMC3553543

[B29] Raichle M. E. (2006). Neuroscience. The brain's dark energy. Science 314, 1249–1250 10.1126/science. 1134405 17124311

[B30] Renart A. De La Rocha J. Bartho P. Hollender L. Parga N. Reyes A. (2010). The asynchronous state in cortical circuits. Science 327, 587–590 10.1126/science.1179850 20110507 PMC2861483

[B31] Roxin A. Hakim V. Brunel N. (2008). The statistics of repeating patterns of cortical activity can be reproduced by a model network of stochastic binary neurons. J. Neurosci. 28, 10734–10745 10.1523/JNEUROSCI.1016-08.2008 18923048 PMC6671336

[B32] Rubinov M. Sporns O. Thivierge J. P. Breakspear M. (2011). Neurobiologically realistic determinants of self-organized criticality in networks of spiking neurons. PLoS Comput. Biol. 7:e1002038 10.1371/journal.pcbi.1002038 21673863 PMC3107249

[B33] Sasaki T. Matsuki N. Ikegaya Y. (2007). Metastability of active CA3 networks. J. Neurosci. 27, 517–528 10.1523/JNEUROSCI.4514-06.2007 17234584 PMC6672786

[B34] Schreiber T. (2000). Measuring information transfer. Phys. Rev. Lett. 85, 461–464 10.1103/PhysRevLett.85.461 10991308

[B35] Shew W. L. Yang H. Yu S. Roy R. Plenz D. (2011). Information capacity and transmission are maximized in balanced cortical networks with neuronal avalanches. J. Neurosci. 31, 55–63 10.1523/JNEUROSCI.4637-10.2011 21209189 PMC3082868

[B36] Shimazaki H. Shinomoto S. (2007). A method for selecting the bin size of a time histogram. Neural Comput. 19, 1503–1527 10.1162/neco.2007.19.6.1503 17444758

[B37] Tang A. Jackson D. Hobbs J. Chen W. Smith J. L. Patel H. (2008). A maximum entropy model applied to spatial and temporal correlations from cortical networks *in vitro*. J. Neurosci. 28, 505–518 10.1523/JNEUROSCI.3359-07.2008 18184793 PMC6670549

[B38] Tauskela J. S. Fang H. Hewitt M. Brunette E. Ahuja T. Thivierge J. P. (2008). Elevated synaptic activity preconditions neurons against an *in vitro* model of ischemia. J. Biol. Chem. 283, 34667–34676 10.1074/jbc.M805624200 18845540 PMC3259903

[B39] Touboul J. Destexhe A. (2010). Can power-law scaling and neuronal avalanches arise from stochastic dynamics? PLoS ONE 5:e8982 10.1371/journal.pone.0008982 20161798 PMC2820096

[B40] van Vreeswijk C. Sompolinsky H. (1998). Chaotic balanced state in a model of cortical circuits. Neural Comput. 10, 1321–1371 9698348 10.1162/089976698300017214

[B41] Vicente R. Wibral M. Lindner M. Pipa G. (2011). Transfer entropy–a model-free measure of effective connectivity for the neurosciences. J. Comput. Neurosci. 30, 45–67 10.1007/s10827-010-0262-3 20706781 PMC3040354

[B42] Wagenaar D. A. Pine J. Potter S. M. (2006). An extremely rich repertoire of bursting patterns during the development of cortical cultures. BMC Neurosci. 7:11 10.1186/1471-2202-7-11 16464257 PMC1420316

